# What Is Typhoid-Pneumonia?

**Published:** 1869-08

**Authors:** 


### What is Typhoid-Pneumonia?

Grissole says that half of the cases of pneumonia occurring in persons with chronic disease of liver and kidneys, and nearly all in those who have chronic disease of the nervous centres assume the adynamic or typhoid form.

That this kind of pneumonia may closely resemble typhus fever, is proved by the fact, now considered to be tolerably well ascertained, that Pinel, one of the ablest systematic writers of France, drew his descriptions of the so-called dynamic and ataxic fevers almost exclusively from cases of pneumonia occurring in old persons. He was physician to the Salpetriere—a hospital for aged people, all of whom were above the age to which typhoid fever of Paris is limited. Pneumonia is now found to be one of the most frequent and fatal diseases of this hospital—and in a large number of cases it presents the same general assemblage of symptoms given by Pinel as characterizing adynamic fever.

Some physicians think that the expression of the countenance is of itself sufficient to characterize typhus fever, but a dull, heavy flush of the face occurs in pneumonia, and the look may be heavy, languid and dull.

Cough and expectoration may be absent, and the patient may not complain of his chest; but, his breathing is generally laborious, and the lips livid, although the skin may be almost natural. In very weak subjects bronchial respiration is absent in at least one-half of all cases; owing partly to the great debility of the patient which prevents him or her from inspiring sufficiently deeply and strongly to produce the tubular sound; and partly to the fact that splenization is more common in the second stage than the firm hepatization which conveys the sound from the air tubes through an almost solid lung.

Hence large crepitation, or muco-crepitant rhonchus is more frequently heard than tubular, or bronchial respiration.

This so-called typhoid pneumonia is also apt to occur during the course of delirium tremens in broken down subjects; the prostation of strength and recumbent position of the patient then favors the congestive engorgement, or sub-inflammation of the posterior lobes of the lungs.—*Medical Gazette.*

